# Smart Bacterial Cellulose–Methylacrylated Chitosan Composite Hydrogel: Multifunctional Characterization for Real-Time pH Monitoring

**DOI:** 10.3390/polym17070914

**Published:** 2025-03-28

**Authors:** Zixian Bao, Jiezheng Liu, Yujia Bi, Guang Zhao

**Affiliations:** 1 State Key Laboratory of Microbial Technology, Institute of Microbial Technology, Shandong University, Qingdao 266237, China; baozixian@sdu.edu.cn (Z.B.);; 2 CAS Key Laboratory of Biobased Materials, Qingdao Institute of Bioenergy and Bioprocess Technology, Chinese Academy of Sciences, Qingdao 266101, China

**Keywords:** bacterial cellulose, methylacrylated chitosan, pH sensor

## Abstract

pH is a critical parameter that influences biochemical and environmental processes. Real-time and accurate pH detection is essential for monitoring health and the environment. Herein, a bacterial cellulose and methylacrylated chitosan (BC-MACS) composite hydrogel was prepared to achieve rapid pH detection. The integration of MACS reduced the crystallinity of pristine BC, with no adverse effects on thermal stability. SEM images validated the fibrous nature of the BC-MACS composite, indicating that MACS was successfully infiltrated into the pores of BC. By incorporating MACS into the BC matrix, the exceptional biocompatibility of BC was maintained, while simultaneously augmenting its mechanical properties. Due to the excellent swelling ability of MACS, the fabricated BC-MACS hydrogel exhibited superior swelling behavior compared to the BC hydrogel, which facilitated the absorption of the solution under test. A BC-MACS pH sensor was fabricated by introducing the pH indicator solution, and the color variation across the pH range (2–12) demonstrated a clear response to pH changes. Therefore, the BC-MACS pH sensor holds potential for use as a visual indicator in a diverse range of applications, especially for health and environmental monitoring.

## 1. Introduction

Biochemical and environmental processes are greatly influenced by pH [1]. The measurement of pH has significant importance in industrial processes, including in chemistry, medicine, biology, and environmental monitoring [2]. To achieve real-time and accurate pH detection, various pH sensors have been developed and are used in a variety of fields, including health monitoring, water quality detection, food packaging, and other processes [1,3,4,5,6,7]. Normally, these pH sensors are composed of a solid support and a colorimetric indicator. The solid support is associated with the sensitivity, response time, reversibility, and reproducibility of the pH sensor [8]. Different natural and synthetic polymers have been used to immobilize a colorimetric indicator to fabricate pH sensors, such as polyethylene [9], chitosan [10,11], starch–polyvinyl alcohol [12], chitosan–polyvinyl alcohol [13], methylcellulose [14], and nanocellulose [8].

Bacterial cellulose (BC), a biopolymer obtained from Gram-negative acetic acid bacteria, possesses satisfactory properties, including high purity, non-toxicity, low immunogenicity, an ultrafine network architecture, high specific surface area, and excellent mechanical strength [15,16]. Based on these properties, BC has been developed to fabricate pH sensors for fish freshness and sweat pH detection [8,17,18,19]. These sensors are prepared by immersing BC films in the colorimetric indicator solution and are subsequently air-dried. Despite the color of these sensors changing with pH, the fibrous structure of pristine BC is reported to collapse upon drying, which can influence the re-swelling ability and may cause the color change distortion [17,20]. Therefore, the re-swelling ability is crucial for the application of a BC-based pH sensor, especially in the fields of health or seawater monitoring. Carboxymethyl cellulose has been incorporated into BC to enhance the re-swelling ability using the undisturbed method, fabricating a colorimetric sensor for sweat pH detection [17]. However, other methods have rarely been reported.

Methylacrylated chitosan (MACS) represents a class of chitosan derivatives synthesized through methacrylate grafting to enhance aqueous solubility and confer the ultraviolet (UV)-initiated cross-linking capability [21]. This modification preserves the inherent biocompatibility of chitosan while introducing advantageous characteristics such as three-dimensional (3D) printability, tunable mechanical properties, and remarkable swelling behavior [21,22]. Therefore, MACS has been proposed to be applied as a drug delivery platform, skin substitute, and tissue-engineering scaffold [21,23,24,25,26]. MACS displays an intriguing swelling behavior, and can rapidly absorb an enormous amount of water (with a swelling ratio higher than 100%) [21,27], which is a gratifying property to incorporate with BC to enhance the water absorption ability.

Herein, MACS was incorporated into BC based on simple immersion and UV irradiation to prepare the BC-MACS hydrogel. The proposed method is straightforward, highly efficient, and necessitates neither special chemicals nor prior modification of the BC. The crystalline structure and surface morphology were characterized using X-ray diffraction (XRD) analysis and scanning electron microscopy (SEM), respectively. The thermal stability, swelling behavior, and mechanical properties were also evaluated. The colorimetric indicator was incorporated into the BC-MACS hydrogel to fabricate the BC-MACS pH sensor, and the color differentiation performance was determined.

## 2. Materials and Methods

### 2.1. Materials

Chitosan (viscosity 72 cps, degree of deacetylation, DD = 90%) was purchased from Laizhou Haili Biological Product Co., Ltd. (Laizhou, China). pH indicator solution was purchased from Beijing Gersion Bio-Technology Co., Ltd. (Beijing, China). Cetyltrimethyl ammonium bromide (CTAB) was purchased from Sigma-Aldrich (St. Louis, MO, USA). High-glucose Dulbecco’s modified Eagle’s medium (DMEM) was obtained from Hyclone (Logan, UT, USA). A CCK-8 kit was purchased from Macklin Biochemical Co., Ltd. (Shanghai, China). All other reagents were of analytical grade and used as received.

### 2.2. Synthesis of MACS and BC

#### 2.2.1. Synthesis of MACS

MACS was synthesized by reacting chitosan with methacrylic anhydride (MA) [26]. In brief, chitosan (1.5 wt%) was dissolved overnight in acetic acid solution (4 wt%). Then, MA at a molar ratio of 100:1 (with respect to chitosan) was added slowly to the chitosan acetic acid solution, which was stirred at 40 °C, 60 rpm, for 12 h, under dark conditions. The resulting production was a milky suspension that was dialyzed (MWCO 8–14 kDa dialysis bag) using deionized water for one week and lyophilized.

#### 2.2.2. Synthesis of BC Pellicles

The BC was produced by *Komagataeibacter sucrofermentans* (*K. sucrofermentans*) fermentation [16]. *K. sucrofermentans* was inoculated in 7% (*v*/*v*) H-S basic medium (containing 25 g L^−1^ of glucose, 5 g L^−1^ of yeast extract, 5 g L^−1^ of peptone, 1.2 g L^−1^ of citric acid monohydrate, and 2.7 g L^−1^ of Na_2_HPO_4_) and cultured at 30 °C for 5 days. The obtained BC pellicles were treated with NaOH solution overnight at 60 °C, and subsequently washed with deionized water. BC pellicles were stored at 4 °C until use.

### 2.3. Preparation of MACS and BC-MACS Hydrogels

MACS was dissolved in deionized water to obtain 20 mg mL^−1^ of homogeneous solution. Subsequently, Irgacure 2959 was added to MACS solution at a final concentration of 0.5 mg mL^−1^. Afterward, the BC pellicles were soaked in the MACS solution for 15 min. The MACS and BC-MACS solutions were exposed to UV light (365 nm) with a light intensity of 5 W cm^−2^ for 15 min to prepare the MACS and the BC-MACS hydrogels [22,28].

### 2.4. Characterization of BC-MACS Hydrogel

#### 2.4.1. FTIR Analysis

The chitosan, MACS (before cross-linking), and air-dried BC-MACS hydrogels (after cross-linking) were characterized using FTIR spectroscopy. The infrared spectra were recorded on an FTIR spectrometer (Nicolet 6700, Thermo Fisher, Waltham, MA, USA) with the frequency range from 4000 to 400 cm^−1^ at a data acquisition rate of 2 cm^−1^ per point [29].

#### 2.4.2. XRD Analysis

The air-dried BC-MACS hydrogel was analyzed with an X-ray diffractometer (D8 Advance, Bruker, Berlin, Germany), operated at 20 kV (CuKα target) with a scanning range of 5–50° and a diffraction rate of 10° min^−1^. The background noise was subtracted following the collection of XRD data [30].

#### 2.4.3. Thermogravimetric Analysis (TGA)

TGA measurements were performed using a thermogravimetric analyzer (TGA2, Mettler Toledo, Greifensee, Switzerland) with a heating rate of 20 °C min^−1^, over a range of 30–800 °C, and under a nitrogen atmosphere (20 mL min^−1^) [26].

#### 2.4.4. SEM

The pristine BC, MACS, and BC-MACS hydrogels were all air-dried and sputter-coated with gold. The surface and cross-section structures were examined using a field emission scanning electron microscope (Quanta 250 FEG, FEI, Hillsboro, OR, USA).

#### 2.4.5. Cytocompatibility

The cytotoxicity of different hydrogels was assessed using a CCK-8 assay against L929 cells. In brief, the MACS, BC, and BC-MACS hydrogels were cultured in the DMEM medium supplemented with 10% (*v*/*v*) FBS (Gibco, Invitrogen, Carlsbad, CA, USA), 100 IU mL^−1^ of penicillin, and 100 μg mL^−1^ of streptomycin (Sigma-Aldrich, Gillingham, UK) at 37 °C for 24 h. The DMEM medium of the overnight cultured L929 cells was replaced with 100 μL of hydrogel extract medium. After 24 h of incubation, CCK-8 solution (10 μL) was added to each well and continuously incubated for 4 h. The absorbance at 450 nm was recorded using a microplate reader. The percentage of viability was calculated as a percentage of the untreated control cells.

### 2.5. Swelling Behavior

The prepared MACS, BC, and BC-MACS hydrogels were weighed (*W*_0_) and then immersed in deionized water at room temperature for 24 h. The weight (*W_t_*) of the swollen hydrogels was determined after removing the excess water. The swelling ratio of the hydrogels was calculated as follows:

(1) $$Swelling\;ratio\;(\%)=\frac{W_{t}-W_{0}}{W_{0}}\times 100$$

### 2.6. Mechanical Properties

The mechanical properties of BC-MACS hydrogels were performed using an Instron 5543 universal testing machine (Instron Corp., Canton, OH, USA). All the samples were air-dried and cut into strips (0.5 × 5 cm^2^). The breaking strength of the samples was conducted with a speed of 30 mm min^−1^ at room temperature. Tensile strength refers to the average of the ultimate stress at failure [30].

### 2.7. Preparation of BC-MACS pH Sensor

CTAB (0.1 wt%) was added to the pH indicator solution (containing methyl red, methyl yellow, thymol blue, bromothymol blue, and phenolphthalein). Subsequently, the BC-MACS hydrogel was immersed in this pH indicator solution for 15 min, and rinsed with deionized water.

The performance of the BC-MACS pH sensor was evaluated by adding different pH buffers (pH 2~11) on the sensor, and the color change was measured in terms of hue angle value using a portable spectrophotometer (Datacolor Check3, Datacolor, Lawrenceville, NJ, USA) [17].

### 2.8. Statistical Analysis

Each experiment was performed independently in triplicate at least. Results were given as mean ± standard deviation (SD). A one-way analysis of variance with Dunnett’s post hoc test was performed. Statistical significance was set as *p* < 0.05.

## 3. Results and Discussion

### 3.1. Preparation and Characterization of BC-MACS Hydrogel

**Scheme 1:**
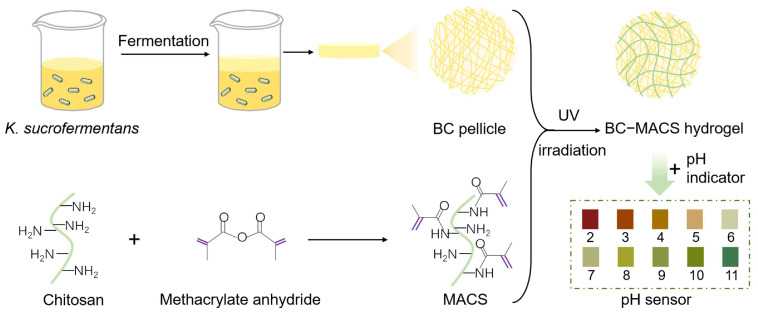
Schematic representation of the preparation process of BC-MACS pH sensor and its application.

**Figure 1:**
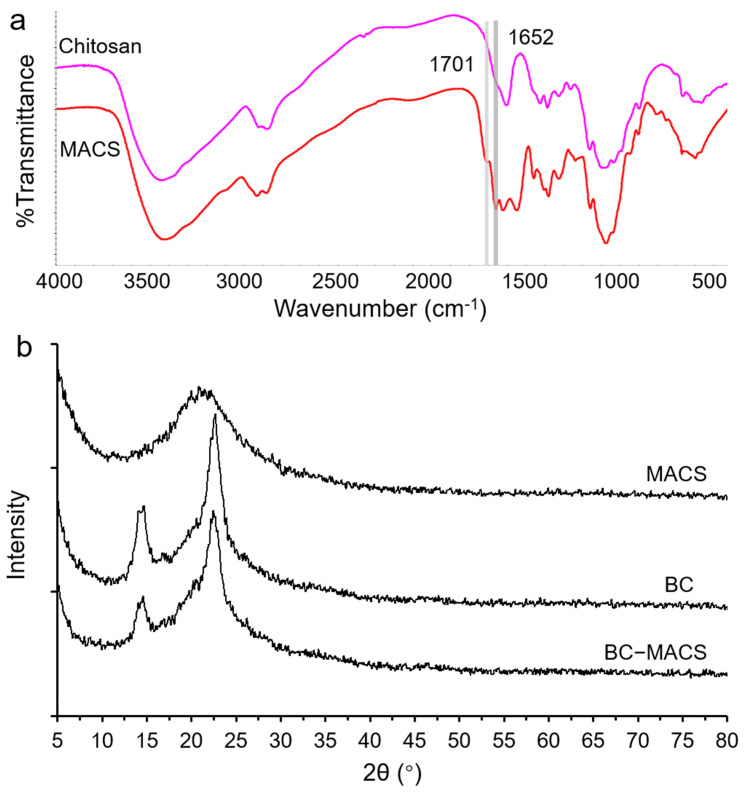
Characterization of MACS and BC-MACS hydrogels: (**a**) FTIR spectra of chitosan and MACS; (**b**) XRD patterns of MACS, BC, and BC-MACS.

In this study, MACS was incorporated into the BC network to fabricate the BC-MACS hydrogel based on UV cross-linking (Scheme 1). Chitosan has abundant amine groups that are available for modification by MA. The result of the FTIR analysis verified the successful synthesis of MACS (Figure 1a). The characterized peaks at 1701 cm^−1^ and 1652 cm^−1^ in the spectrum of MACS were associated with a C=O group and C=C group, respectively, belonging to MA [29]. After cross-linking, the disappearance of the C=C group peak centered at 845 cm^−1^ indicated that MACS still owned high reactivity when incorporated into the BC network (Figure S1) [27]. The result suggested that the BC-MACS semi-interpenetrating network was formed after UV cross-linking. XRD analysis was performed to investigate the crystalline structure of the BC-MACS. As shown in Figure 1b, pure air-dried MACS hydrogel shows an amorphous state, characterized by the presence of a broad peak in the 2θ scattering angle range from 17° to 25°. The pristine BC and BC-MACS hydrogel exhibits two characteristic absorption peaks at 14.5° and 22.6°, which were assigned to the (110) and (200) planes of the cellulose form I-β crystal [16,31,32]. However, the intensity of these two diffraction peaks in the BC-MACS group was slightly lower than that of the BC group, indicating that the crystallinity of BC was slightly decreased after the integration of MACS [33].

**Figure 2:**
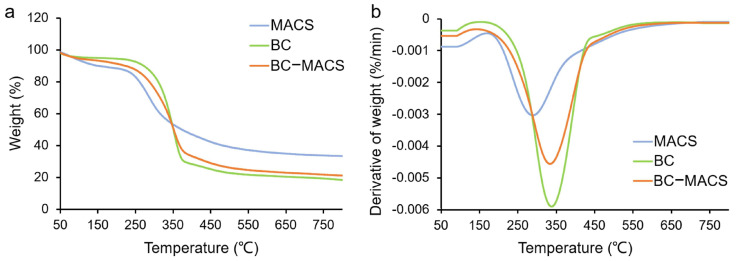
Thermal stability of MACS, BC, and BC-MACS: (**a**) TGA profiles; (**b**) DTG curves.

The thermal stability of the BC-MACS hydrogel was evaluated by TGA (Figure 2). Pure MACS exhibited a weight loss of 47% in the temperature range of 244–408 °C. The pristine BC exhibited a more substantial decomposition profile, with a 66% mass loss occurring between 246 and 397 °C, indicative of significant thermal decomposition. This behavior is attributed to the breakdown of the cellulose backbone structure. For the BC-MACS, the degradation (62% weight loss) occurred in the temperature range of 214–412 °C, suggesting a broader decomposition temperature range compared to individual components. To further characterize thermal properties, differential thermogravimetry (DTG) curves were analyzed (Figure 2b). The maximum thermal decomposition temperature of pure MACS was 289 °C, while pristine BC exhibited a higher peak temperature of 341 °C. This enhanced thermal stability in BC is associated with its crystalline structure, as confirmed by the X-ray diffraction (XRD) patterns in Figure 1. Conversely, MACS forms an amorphous matrix, which correlates with its lower decomposition temperature. The thermal decomposition temperature of BC-MACS was 335 °C, which had no significant difference from that of pristine BC. The result indicated that the crystalline BC component dominated the thermal behavior in the composite system.

**Figure 3:**
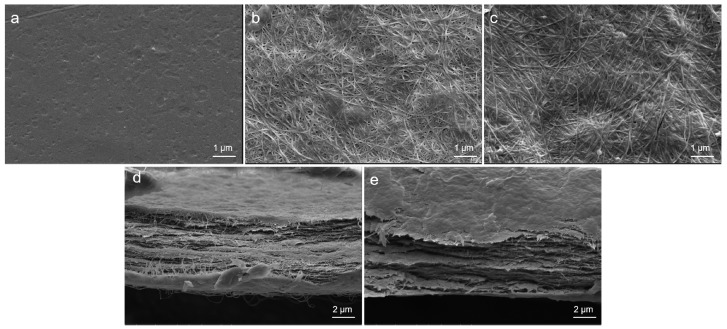
SEM images of different air-dried hydrogels. (**a**–**c**) Surface morphology of MACS (**a**), BC (**b**), and BC-MACS (**c**). The scale bar corresponds to 1 μm. (**d**,**e**) Cross-section morphology of BC (**d**) and BC-MACS (**e**). The scale bar corresponds to 2 μm.

The surface and cross-section morphology of the BC-MACS were imaged by SEM (Figure 3). The pure MACS hydrogel (after UV cross-linking) shows a smooth surface structure (Figure 3a). Both BC and BC-MACS exhibit fibrous networks. For the BC samples, the fibers are packed and tiny pores can be observed in the margin of the stacked fibers (Figure 3b). For the BC-MACS samples, only a few tiny pores can be observed (Figure 3c), which might be due to the permeation of the incorporated MACS solution into these pores. MACS was subsequently cross-linked in situ by UV irradiation to form the hydrogel, leading to the disappearance of pores. However, compared to the BC samples, the stacked form of fibers, fiber size, and nanofibrous structure in BC-MACS were not influenced by the incorporation of MACS. Compared to the surface morphology, the internal morphology shows no significant difference between the BC and BC-MACS groups (Figure 3d,e), indicating that the incorporated MACS was mainly deposited on the surface and only a small proportion penetrated into the interior.

**Figure 4:**
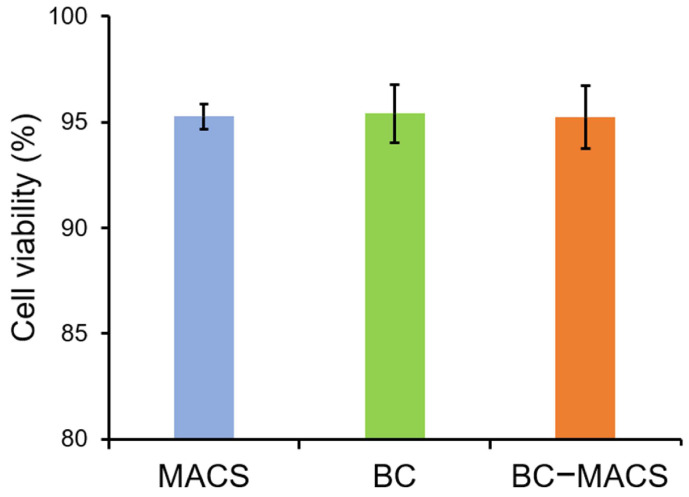
The cytotoxicity of MACS, BC, and BC-MACS hydrogels.

A CCK-8 assay was used to evaluate the cytotoxicity of the BC and MACS hydrogels (Figure 4). The relative cell viability of the MACS, BC, and BC-MACS hydrogels was all higher than 90%, indicating their excellent biocompatibility. All of the groups showed no significant difference in their cell viability, demonstrating that the incorporation of MACS could not alter the biocompatibility of pristine BC.

### 3.2. Swelling Behavior

**Figure 5:**
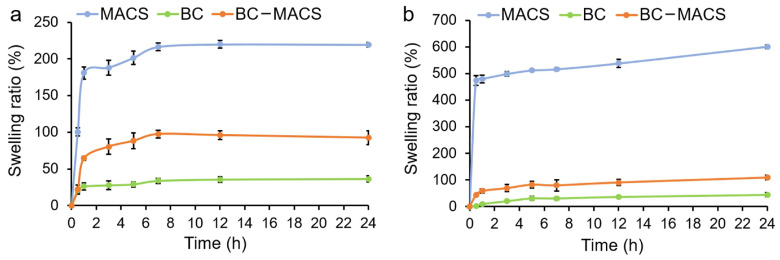
Swelling ratios of MACS, BC, and BC-MACS hydrogels: (**a**) wet hydrogels; (**b**) air-dried hydrogels.

In order to incorporate the pH indicator, the fabricated hydrogel should rapidly absorb large amounts of solution. The swelling behavior of the MACS hydrogel was rapid and achieved the swelling balance within 1 h (Figure 5a). The swelling ratio almost reached 216% for the MACS hydrogel. However, the swelling ratio of the BC hydrogel was much lower than that of the MACS hydrogel, and the maximum swelling ratio was 36.4 ± 4.0%. The swelling behavior of the BC-MACS hydrogel was intervening between the MACS and BC hydrogels, and the swelling ratio could reach around 64% within 1 h. Due to the incorporation of MACS, the swelling behavior was enhanced 2-fold, facilitating the absorption of the pH indicator into the BC-MACS hydrogels. For air-dried hydrogels, the change trend of the swelling ratio among different groups (MACS, BC, and BC-MACS) was similar to that of the corresponding wet hydrogels (Figure 5b). Air-dried MACS hydrogel could absorb more solution compared to the wet hydrogel, and the final swelling ratio was 601.9 ± 6.7%. In addition, the swelling behavior of MACS occurred rapidly, and the swelling balance was achieved within 0.5 h. However, the air-dried BC hydrogel exhibited a lower swelling ratio (1.3 ± 0.6%) in comparison to wet hydrogel within 0.5 h, although the final swelling ratio displayed no significant difference between the air-dried and wet BC hydrogels. For the air-dried BC-MACS hydrogel, swelling balance was also reached within 0.5 h, and the swelling ratio (42.9 ± 0.3%) was considerably higher than that of the air-dried BC hydrogel. Therefore, the incorporation of MACS enhanced the swelling property of the BC hydrogel. The rapid and massive absorption of solution was crucial for the BC-MACS hydrogel to realize its function, especially as a pH sensor.

### 3.3. Mechanical Properties

**Figure 6:**
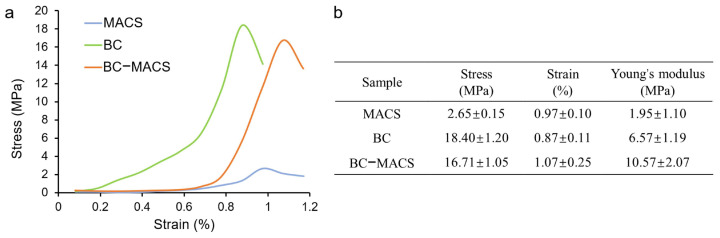
Mechanical properties of different hydrogels: (**a**) stress–strain curves; (**b**) profiles of fracture stress, strain, and Young’s modulus of different hydrogels.

The tensile testing was performed to evaluate the mechanical performance of the air-dried BC-MACS (Figure 6a). The integration of MACS has no significant influence on the mechanical properties of BC. The fracture stress was 18.40 ± 1.20 MPa for BC, consistent with previously reported values for BC [34]. In contrast, MACS demonstrated a lower fracture stress of 2.65 ± 0.15 MPa, which can be attributed to its amorphous structural configuration (as evidenced by the XRD analysis in Figure 1b) compared to the unique 3D nanofibrillar network of BC [34]. The integration of MACS with BC was fabricated by a facile matrix immersion methodology coupled with UV cross-linking. This approach preserved the mechanical integrity of BC, as evidenced by the comparable fracture stress between the BC hydrogel (18.40 ± 1.20 MPa) and BC-MACS hydrogel (16.71 ± 1.05 MPa). These findings corroborate previous research demonstrating that non-cross-linked collagen-BC blends exhibit negligible changes in mechanical performance [34]. The fracture strain of BC-MACS (1.07 ± 0.25%) was slightly enhanced compared to BC (0.87 ± 0.11%) (Figure 6b). The Young’s modulus of BC (6.57 ± 1.19 MPa) was increased to 10.57 ± 2.07 MPa (BC-MACS) when incorporated with MACS, indicating an enhancement in stiffness in BC-MACS.

### 3.4. BC-MACS-Based Colorimetric pH Sensor

**Figure 7:**
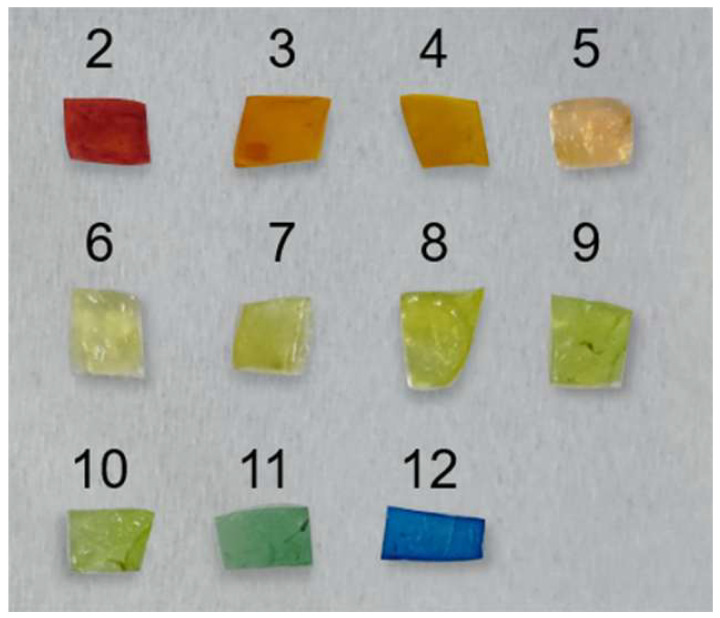
Color changes in BC-MACS pH sensor tested with different pH buffers.

CTAB was introduced in the BC-MACS pH sensor to enhance the stability of the pH indicator inside the BC-MACS hydrogel via the electrostatic interactions between the positively charged CTAB and negatively charged indicator dyes [17,35,36]. The BC-MACS hydrogel exhibited a fast response to different pH buffers with the color change. The color change occurred immediately when adding the pH buffer (<3 s) and could be differentiated by the naked eye. The color of the pH indicator appeared red at a low pH (pH 2), then turned to orange and green when the pH was 3 to 11, and turned to blue at pH 12 (Figure 7). The hue angle of the calibration plot displayed a good correlation coefficient of the determination (R^2^ = 0.9686) (Figure S2a).

BC membranes have been adapted into film or paper-based sticker sensors for food freshness detection [8,18,19], where high water absorptivity is not a critical requirement. The BC-MACS hydrogels developed in this study exhibit exceptional toughness and water adsorption capacity, rendering them highly stable in environments with substantial moisture (such as sweat, urine, and tears) and well-suited for use as wearable biosensors, outperforming traditional paper test strips in terms of conformability. Moreover, the BC-MACS hydrogel undergoes a noticeable color change with just a minute sample volume of 10 μL, significantly less than the 50 μL required by a previously reported textile-based pH sensor [36]. Additionally, the BC-MACS hydrogel boasts a broader range of detection accuracy (pH 2–11) compared to the BC/carboxymethyl cellulose pH sensor (pH 4–9) [17] or the fluorescent-probe-incorporated BC sensor (pH 3–7.5) [37], thereby expanding its potential applications across diverse fields.

A BC pH sensor was also prepared with the same method as the BC-MACS pH sensor, in which deionized water was used instead of MACS solution. However, the color difference in the BC pH sensor was not distinguishable. The color was similar in the pH range of 3–10 (Figure S2b) when observed by the naked eye. The reason might be due to the relatively poor swelling behavior of BC, in which the small amount of the fluid sample could not absolutely interact with the pH indicator to induce the color change [17]. A MACS pH sensor was also prepared. Although the color change in the MACS pH sensor was similar to the BC-MACS pH sensor, the poor mechanical strength led to it easily breaking. Based on the above results, BC-MACS could be used as an excellent pH sensor in a wide range of applications.

## 4. Conclusions

This study introduced a novel BC-MACS hydrogel created by integrating MACS into the BC network through UV cross-linking. XRD analysis revealed a minor crystallinity reduction upon MACS incorporation, with no adverse effects on thermal stability. SEM imaging confirmed the fibrous BC-MACS structure with MACS permeation into the BC pores. The integration of MACS into the BC matrix preserved the exceptional biocompatibility of BC while enhancing its swelling behavior and mechanical properties, especially stiffness. Notably, the BC-MACS hydrogel exhibited rapid swelling kinetics, making it essential for pH sensor applications. The fabricated BC-MACS pH sensor, stabilized by CTAB, demonstrated swift responsiveness to various pH buffers (ranging from pH 2 to 12) with distinct color changes. Therefore, the BC-MACS hydrogel holds promise as a versatile pH sensor for real-time monitoring in diverse applications such as biomedical devices (e.g., wound dressings) and environmental pollution detection systems. Its biocompatibility and mechanical durability further broaden its potential use in wearable biosensors or implantable diagnostic tools.

## Data Availability

The original contributions presented in this study are included in the article/Supplementary Materials. Further inquiries can be directed to the corresponding author.

## Supplementary Materials

The following supporting information can be downloaded at https://www.mdpi.com/article/10.3390/polym17070914/s1, Figure S1: FTIR spectra of BC, MACS, and BC-MACS hydrogels (after cross-linking); Figure S2: A calibration plot of the BC-MACS pH sensor over a pH range of 3–10 (a) and color changes in the BC pH sensor tested with different pH buffers (b).
