# Supplementary material for: Smart Bacterial Cellulose–Methylacrylated Chitosan Composite Hydrogel: Multifunctional Characterization for Real-Time pH Monitoring
Source: Polymers (Basel). 2025 Mar 28;17(7):914. doi: 10.3390/polym17070914 (PMC11991319; doi:10.3390/polym17070914)
Supplement: Supplementary file 1 [file polymers-17-00914-s001.zip › polymers-3514650-supplementary.pdf]

Supplementary Materials

Smart bacterial cellulose–methylacrylated chitosan composite hydrogel: Multifunctional characterization for real-time pH monitoring

Zixian Bao [1], Jiezheng Liu [1,2], Yujia Bi [1] and Guang Zhao [1,*]

[1] State Key Laboratory of Microbial Technology and Institute of Microbial Technology, Shandong University, Qingdao 266237, China.

[2] CAS Key Laboratory of Biobased Materials, Qingdao Institute of Bioenergy and Bioprocess Technology, Chinese Academy of Sciences, Qingdao, 266101, China.

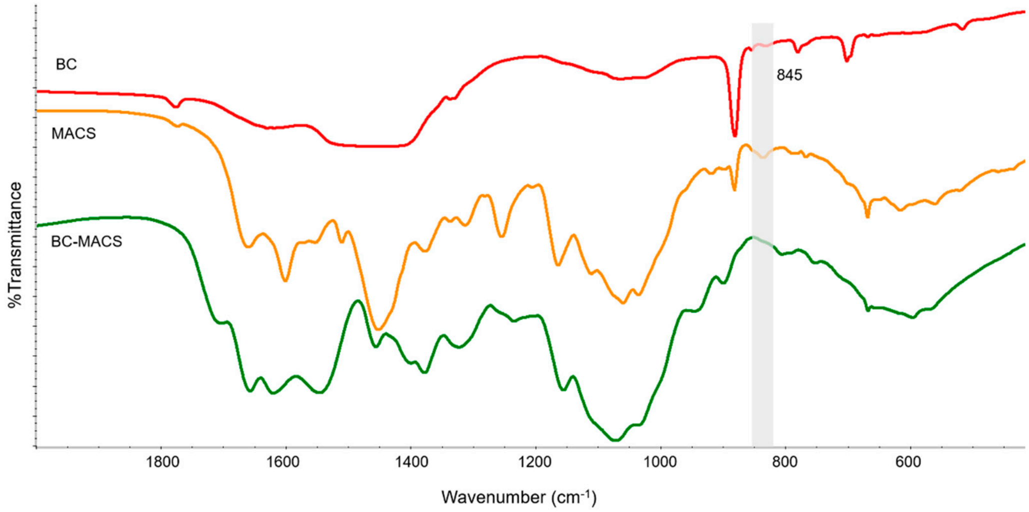

Figure S1. FTIR spectra of BC, MACS, and BC-MACS hydrogels (after crosslinking).

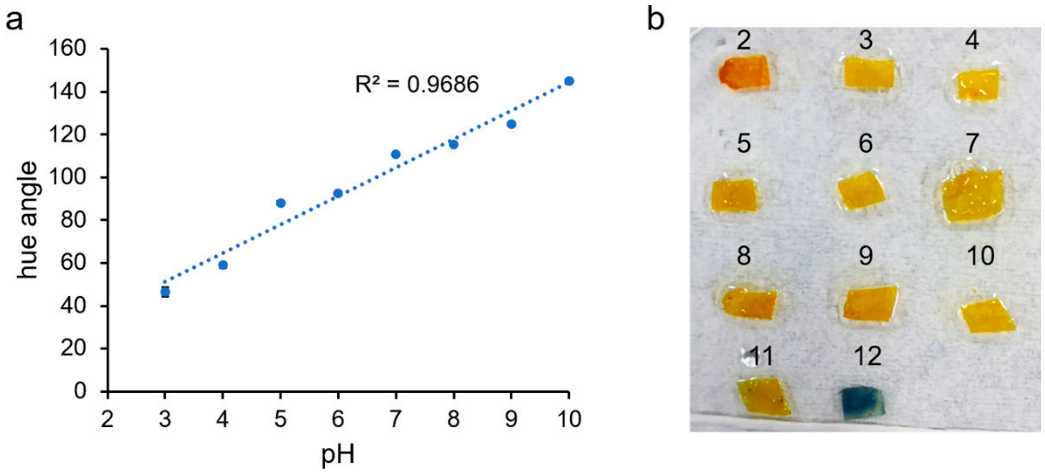

Figure S2. A calibration plot of the BC-MACS pH sensor over a pH range of 3–10 (a); and color changes in the BC pH sensor tested with different pH buffers (b).
